# Interferon Lambda Regulates Cellular and Humoral Immunity in Pristane-Induced Lupus

**DOI:** 10.3390/ijms222111747

**Published:** 2021-10-29

**Authors:** Tom Aschman, Sandra Schaffer, Stylianos Iason Biniaris Georgallis, Antigoni Triantafyllopoulou, Peter Staeheli, Reinhard E. Voll

**Affiliations:** 1 Department of Rheumatology and Clinical Immunology, Medical Center—University of Freiburg, Faculty of Medicine, University of Freiburg, 79106 Freiburg im Breisgau, Germany; s.schaffer@posteo.de (S.S.); iason.biniaris@drfz.de (S.I.B.G.); antigoni.triantafyllopoulou@charite.de (A.T.); 2 Department of Neuropathology, Charité–Universitätsmedizin Berlin, Corporate Member of Freie Universität Berlin, Humboldt-Universität zu Berlin and Berlin Institute of Health, 10117 Berlin, Germany; 3 Innate Immunity in Rheumatic Diseases, Deutsches Rheuma-Forschungszentrum, 10117 Berlin, Germany; 4 Department of Rheumatology and Clinical Immunology, Charité—Universitätsmedizin Berlin, 10117 Berlin, Germany; 5 Institute of Virology, Medical Center—University of Freiburg, Faculty of Medicine, University of Freiburg, 79104 Freiburg im Breisgau, Germany; peter.staeheli@uniklinik-freiburg.de; 6 Center for Chronic Immunodeficiency (CCI), Medical Center—University of Freiburg, Faculty of Medicine, University of Freiburg, 79106 Freiburg im Breisgau, Germany

**Keywords:** SLE, lupus, type III interferons, *Ifnlr1*, interferon lambda, pristane, autoimmunity

## Abstract

A pivotal role of type I interferons in systemic lupus erythematosus (SLE) is widely accepted. Type III interferons (IFN-λ) however, the most recently discovered cytokines grouped within the interferon family, have not been extensively studied in lupus disease models yet. Growing evidence suggests a role for IFN-λ in regulating both innate and adaptive immune responses, and increased serum concentrations have been described in multiple autoimmune diseases including SLE. Using the pristane-induced lupus model, we found that mice with defective IFN-λ receptors (*Ifnlr1*^−/−^) showed increased survival rates, decreased lipogranuloma formation and reduced anti-dsDNA autoantibody titers in the early phase of autoimmunity development compared to pristane-treated wild-type mice. Moreover, *Ifnlr1*^−/−^ mice treated with pristane had reduced numbers of inflammatory mononuclear phagocytes and cNK cells in their kidneys, resembling untreated control mice. Systemically, circulating B cells and monocytes (CD115^+^Ly6C^+^) were reduced in pristane-treated *Ifnlr1*^−/−^ mice. The present study supports a significant role for type III interferons in the pathogenesis of pristane-induced murine autoimmunity as well as in systemic and renal inflammation. Although the absence of type III interferon receptors does not completely prevent the development of autoantibodies, type III interferon signaling accelerates the development of autoimmunity and promotes a pro-inflammatory environment in autoimmune-prone hosts.

## 1. Introduction

Systemic lupus erythematosus (SLE) is an autoimmune disorder presenting with a large variety of clinical symptoms and mostly affects women of childbearing age [1,2,3]. One key element in the pathogenesis of SLE is the loss of tolerance against nuclear self-antigens and the ensuing development of anti-nuclear autoantibodies. This appears to result from an unfavorable combination of genetic variants that affect one or multiple mechanisms that usually prevent the development of autoimmunity [4]. The disturbance of distinct B cell tolerance checkpoints can lead to the emergence, multiplication and maintenance of autoantibodies, which usually precede the clinical manifestations of disease [5,6]. Autoantibodies contribute to multiple disease manifestations, including autoimmune hemolytic anemia, immune thrombocytopenia, antiphospholipid syndrome, lupus nephritis and neonatal lupus. Long-lived plasma cells producing pathogenic autoantibodies appear to be major contributors to the pathogenesis of SLE and might be responsible for treatment-refractory disease courses [7,8]. The progressive multi-organ inflammation seen in lupus patients leads to cellular damage and the increased exposure of nuclear antigens, resulting in a self-perpetuating, vicious circle [9].

A crucial role of type I IFN in disease progression has been suspected since the 1980s and was meanwhile corroborated in humans as well as in various animal models [10,11,12]. Administration of an adenovirus encoding IFN-α or the administration of double-stranded RNA accelerates and worsens several parameters of disease activity in murine lupus models, and this effect was mostly attributed to higher autoantibody production and enhanced glomerular immune complex deposition [13,14,15,16,17]. Most importantly, the application of IFN-α can cause drug-induced lupus in humans. Vice versa, blockade of the type I IFN pathway using anifrolumab, an antibody against the type I IFN receptor, ameliorated SLE in clinical trials [18] and was recently approved by the FDA for the treatment of SLE. 

The role of type III IFNs in SLE, however, is not well understood. They are the most recently discovered members of the interferon family and comprise four distinct but closely related cytokines, named IFN-λ1 (or IL29), IFN-λ2 (or IL28A), IFN-λ3 (or IL28B) and IFN-λ4. Type III IFNs are distantly related to type I IFNs and IL-10 [19,20,21,22]. All four type III IFNs signal through a receptor complex comprising two subunits, namely IFNLR1 (or CRF2-12) and a second subunit, IL-10R2 (or CRF2-4) [20,23]. Whereas IL-10R2 is constitutively expressed on a wide variety of human cell lines and tissues, IFNLR1 is expressed at high levels on epithelial cells and at low levels on some immune cells [24]. As with type I IFNs, signaling of type III IFNs occurs through the JAK-STAT pathway [20,23]. 

Several independent studies found higher serum concentrations of type III IFNs in patients with SLE when compared to healthy controls and even higher concentrations in patients with active SLE. Type III IFN levels correlated with SLE Disease Activity Index scores and anti-double-stranded DNA (dsDNA) autoantibody titers [25,26,27,28,29]. A role for IFN-λ in murine SLE was recently reported in a TLR7-induced lupus mouse model [30]. *Ifnlr1*-deficient mice exposed to the TLR7 agonist imiquimod showed signs of reduced myeloid expansion and T cell activation, and signs of reduced skin and kidney inflammation with less immune complexes deposited. Interestingly, the authors reported that there was no reduction in autoantibody titers [30]. Chronic application of imiquimod induces skin inflammatory disease, which results in systemic autoimmunity following the likely disruption of the epidermal barrier, and is thus used as a murine model for human psoriasis, but also for SLE because of the observed development of anti-dsDNA autoantibodies and systemic organ damage including glomerulonephritis [31,32]. In contrast, lupus nephritis patients do not always have skin disease. 

To address this conundrum, we used the well-established pristane-induced model of murine lupus in *Ifnlr1*-deficient mice. Lupus-like disease can be induced by a single intra-peritoneal injection of pristane (2,6,10,14-tetramethylpentadecane TMPD), a substance naturally occurring in mineral oil and plants. Its administration provokes polyclonal hypergammaglobulinemia and the production of lupus-associated autoantibodies such as anti-dsDNA, anti-RNP, anti-Sm and anti-U1RNP in any inbred mouse strain, with certain strain-dependent differences regarding antigen specificity [33,34]. Autoantibodies usually appear in the serum two to three months after the injection of pristane [35]. C57BL/6 mice treated with pristane only develop a mild mesangial disease [36,37]. Pristane can also cause pulmonary capillaritis, with some of the mice dying from alveolar hemorrhage within the first three weeks after pristane injection [38,39]. 

Pristane-induced lupus shows a clear type I interferon signature—similar to human lupus—mediated by the TLR7/MyD88 pathway and it was found that—in contrast to human SLE, where plasmacytoid dendritic cells have been identified as the main type I IFN-secreting cells—immature peritoneal Ly6C^high^ monocytes adopt this function in pristane-induced lupus [40,41,42]. The generation of ectopic lymphoid tissue in the peritoneum of mice treated with pristane—unlike imiquimod-induced lupus—may critically contribute to the excessive type I IFN and—at least in part—to autoantibody production, thus mimicking human SLE [43,44]. Lastly, female mice are more prone to developing lupus after pristane treatment than male mice [45]. To our knowledge, the role of type III IFN has not been investigated in this murine lupus model yet. 

To study the role of type III IFN in autoimmunity and lupus-like disease, we injected pristane into mice deficient in functional type III IFN receptors (*Ifnlr1*^−/−^) and wild-type controls.

## 2. Results

### 2.1. Pristane-Treated Ifnlr1^−/−^ Mice Show Reduced Numbers of Lipogranulomas and a Better Survival Rate

**Figure 1:**
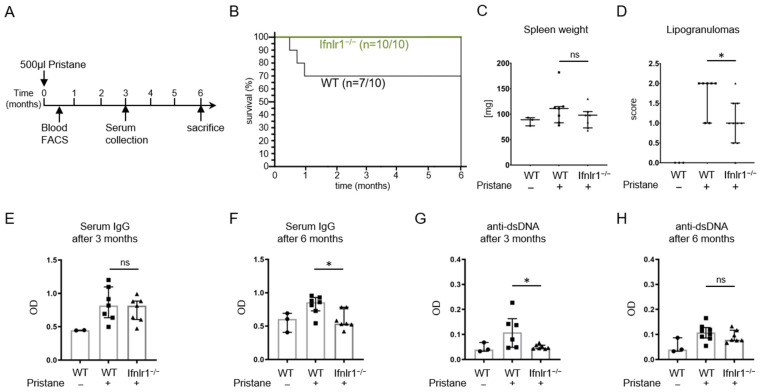
Experimental design, survival and autoimmunity parameters. (**A**) WT and *Ifnlr1*^−/−^ mice were intraperitoneally injected with 500 µL of pristane and sacrificed after 6 months. Blood and serum were collected at 2 weeks and 3 and 6 months after pristane injection. (**B**) After injection with pristane, all *Ifnlr1*^−/−^ mice survived until the 6-month time point, whereas 70% (n = 7/10) of WT mice died within the first month after injection. (**C**) Spleen weight at sacrifice. (**D**) Semi-quantitative scoring of lipogranulomas showed decreased lipogranuloma formation in *Ifnlr1*^−/−^ mice (0 = none; 1 = low; 2 = moderate and 3 = high numbers of lipogranulomas per mouse. (**E**,**F**) Total IgG levels were determined by ELISA at 3 and 6 months after pristane treatment. (**G**,**H**) Anti-dsDNA-IgG levels were determined at 3 and 6 months after pristane treatment by ELISA. Each symbol represents an individual sample and all samples were included. Bars represent median with interquartile range. Statistical analyses to compare WT + pristane vs. *Ifnlr1*^−/−^ + pristane were performed using Mann–Whitney U test. * = *p* < 0.05; ns = not significant. Values of WT without pristane are shown as reference. Findings were confirmed in at least one other independent experiment.

*Ifnlr1*^+/+^ (WT) and *Ifnlr1*^−/−^ mice were intraperitoneally injected with 500 µL of pristane and sacrificed after 6 months (Figure 1A). All of the *Ifnlr1*^−/−^ mice (n = 10/10) survived, whereas 70% (n = 7/10) of the WT mice died within the first month after injection (Figure 1B). The survival rate of WT mice is in line with previous studies using the pristane-induced lupus model on mice with a C57BL/6 background [39]. 

Pristane-treated C57BL/6 mice were previously reported to develop moderate splenomegaly [35]. In our study, only a subset of mice treated with pristane developed splenomegaly and no significant difference was observed between WT and *Ifnlr1*^−/−^ mice (Figure 1C). 

Intraperitoneal injections of pristane are known to result in local chronic inflammation and the formation of lipogranulomas within the peritoneal cavity, a form of ectopic tertiary lymphoid tissue [44]. Semi-quantitative scoring of lipogranulomas showed significantly decreased numbers in *Ifnlr1*^−/−^ mice (Figure 1D). Intraperitoneal pristane injection usually results in the development of hypergammaglobulinemia and lupus-associated antinuclear and anti-dsDNA autoantibodies [33,34]. 

Serum was collected at two distinct time points after pristane injection and total IgG levels were determined by ELISA. Interestingly, no difference was found at the 3-month time point, when WT and *Ifnlr1*^−/−^ mice showed similar levels of reactive hypergammaglobulinemia. However, three months later, at the time of euthanization of the same mice, total IgG levels were significantly reduced in the *Ifnlr1*^−/−^ mice compared to WT controls (Figure 1E,F). 

Serum titers of anti-dsDNA-IgG autoantibodies were determined by ELISA 3 months and 6 months after pristane treatment. IgG antibodies to dsDNA were significantly reduced in *Ifnlr1*^−/−^ mice at 3 months but not at 6 months when compared to WT mice (Figure 1G,H). These data suggest that type III IFN accelerates but is not absolutely required for the development of autoimmunity.

### 2.2. Trend toward Reduced Numbers of Antibody-Secreting Cells in the Spleens and Kidneys of Pristane-Treated Ifnlr1^−/−^ Mice

**Figure 2:**
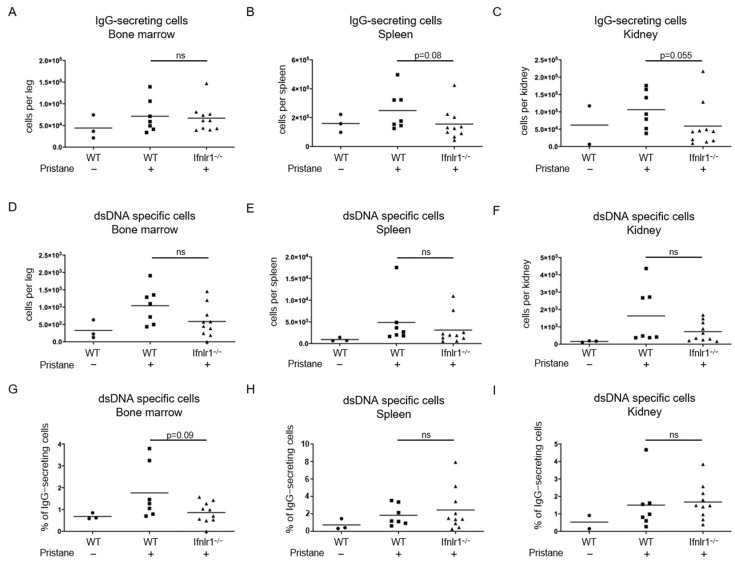
Deficient type III IFN signaling results in a trend towards reduced numbers of antibody-secreting cells in the spleen and kidneys of pristane-treated mice. Numbers of IgG-secreting cells in (**A**) bone marrow, (**B**) spleen and (**C**) kidneys and numbers of anti-dsDNA-IgG-secreting cells in (**D**) bone marrow, (**E**) spleen and (**F**) kidneys at 6 months after pristane treatment were determined by ELISPOT assay. (**G**–**I**) Percentage of anti-dsDNA-IgG-secreting cells of total IgG-secreting cells in indicated tissues. Each symbol represents an individual sample and all samples were included. Bars represent group mean. Statistical analyses to compare WT + pristane vs. *Ifnlr1*^−/−^ + pristane were performed using Mann–Whitney U test; ns = not significant. Values of WT without pristane are shown as reference.

Absolute numbers of total IgG as well as anti-dsDNA-IgG antibody-secreting cells were measured by ELISPOT assay in the bone marrow, spleen and kidneys at 6 months after pristane treatment (Figure 2A–F). Although the differences did not reach statistical significance, there was a clear trend toward reduced numbers of antibody-secreting cells (ASC) in the spleens and kidneys of pristane-treated *Ifnlr1*^−/−^ mice compared to WT mice in two independent experiments (Figure 2B,C). When calculating the proportion of anti-dsDNA-IgG-secreting cells among all ASC, there was also a trend to reduced numbers in *Ifnlr1*^−/−^ mice (Figure 2G–I).

### 2.3. Interferon Lambda Promotes Replacement of Tissue Resident Macrophages with Inflammatory Monocytes

**Figure 3:**
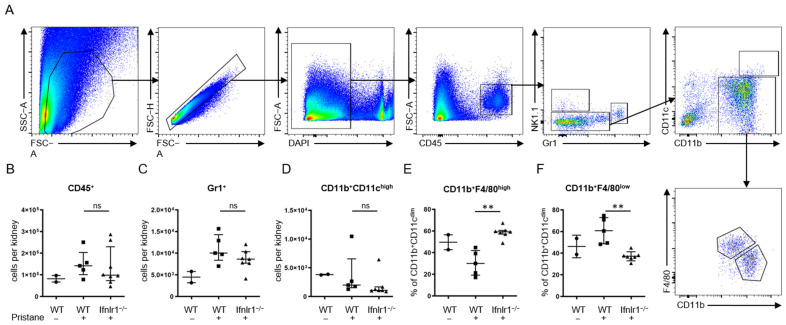
Interferon lambda promotes replacement of tissue-resident macrophages with inflammatory monocytes. (**A**) Flow cytometry gating strategy. Total cell numbers per kidney of CD45^+^ leukocytes (**B**), GR1^+^ neutrophils (**C**), CD11b^+^CD11c^high^ conventional dendritic cells (cDCs) (**D**) and F4/80 surface expression on CD11b^+^Gr1^−^CD11c^−^ monocytes (**E**,**F**) were determined by flow cytometry at 6 months after pristane treatment. Each symbol represents an individual sample; bars represent median with interquartile range. For some mice, not enough tissue was available for flow cytometry. Lin = Lineage (CD3, CD5, CD19, Ly6G). Statistical analyses to compare WT + pristane vs. *Ifnlr1*^−/−^ + pristane were performed using Mann–Whitney U test. ** = *p* < 0.005; ns = not significant. Values of WT without pristane are shown as reference. Findings were confirmed in at least one other independent experiment.

Immune cell populations in the kidney were analyzed by flow cytometry at 6 months after pristane treatment. No difference was found in the total numbers of CD45^+^ leukocytes, Gr1^+^ neutrophils and CD11b^+^CD11c^high^ conventional dendritic cells (cDCs) (Figure 3B–D). However, renal CD11b^+^Gr1^−^CD11c^−^ mononuclear phagocytes of pristane-treated *Ifnlr1*^−/−^ mice showed a clearly distinct phenotype. While pristane suppressed the numbers of mononuclear phagocytes expressing higher levels of F4/80 (bona fide tissue-resident macrophages) in WT mice, pristane-injected *Ifnlr1*^−/−^ mice had frequencies of F4/80^hi^ mononuclear phagocytes, similar to non-injected mice (Figure 3E,F), suggesting that the renal tissue-resident macrophage numbers are maintained at homeostatic levels in the absence of IFN-λ signaling.

### 2.4. After Pristane Treatment, Ifnlr1^−/−^ Mice Show Reduced Numbers of Conventional NK Cells in the Kidney Compared to Wild-Type Mice

**Figure 4:**
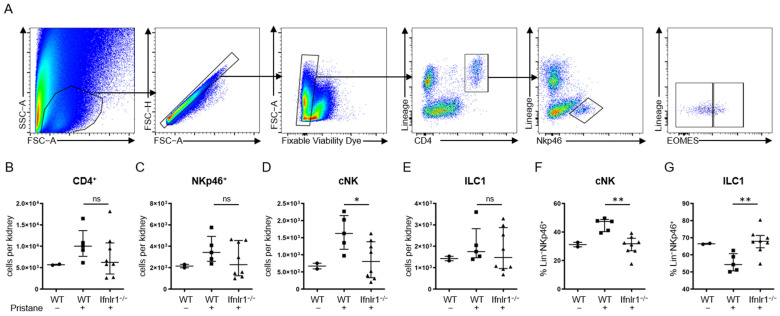
*Ifnlr1*^−/−^ mice show reduced numbers of cNK cells in the kidney after pristane treatment. (**A**) Flow cytometry gating strategy. Total cell numbers per kidney of CD4^+^ cells (**B**) NKp46^+^Lin^−^ NK cells (**C**), NKp46^+^Lin^−^Eomes^+^ conventional NK cells (**D**,**F**) and NKp46^+^Lin^−^Eomes^−^ ILC1 cells (**E**,**G**) were determined by flow cytometry at 6 months after pristane treatment. Lin = Lineage (CD3, CD5, CD19, Ly6G). Each symbol represents an individual sample, bars represent median with interquartile range. For some mice, not enough tissue was available for flow cytometry. Statistical analyses to compare WT + pristane vs. *Ifnlr1*^−/−^ + pristane were performed using Mann–Whitney U test. * = *p* < 0.05; ** = *p* < 0.005; ns = not significant. Values of WT without pristane are shown as reference. Findings were confirmed in at least one other independent experiment.

Total cell numbers per kidney of CD4^+^ T cells, NKp46^+^Lin^−^ cells, NKp46^+^Lin^−^Eomes^+^ conventional NK cells and NKp46^+^Lineage (CD3, CD5, CD19, Ly6G)^−^ Eomes^−^ ILC1 cells were determined by flow cytometry at 6 months after pristane treatment (Figure 4). While no differences in the numbers of CD4^+^ T cells and NKp46^+^Lineage^−^ cells were found in *Ifnlr1*^−/−^ mice compared to WT controls (Figure 4B,C), the numbers of NKp46^+^Lin^−^Eomes^+^ conventional NK cells were significantly reduced (Figure 4F). IFNLR1 deficiency, in addition, reversed the proportion of Eomes^+^ and Eomes^−^ cells to similar levels as seen in untreated control mice (Figure 4F,G), suggesting that IFN-λ signaling promotes the recruitment of NK cells to the kidneys in murine lupus.

### 2.5. Ifnlr1^−/−^ Mice Display Reduced Numbers of B Cells in the Spleen after Pristane Treatment

**Figure 5:**
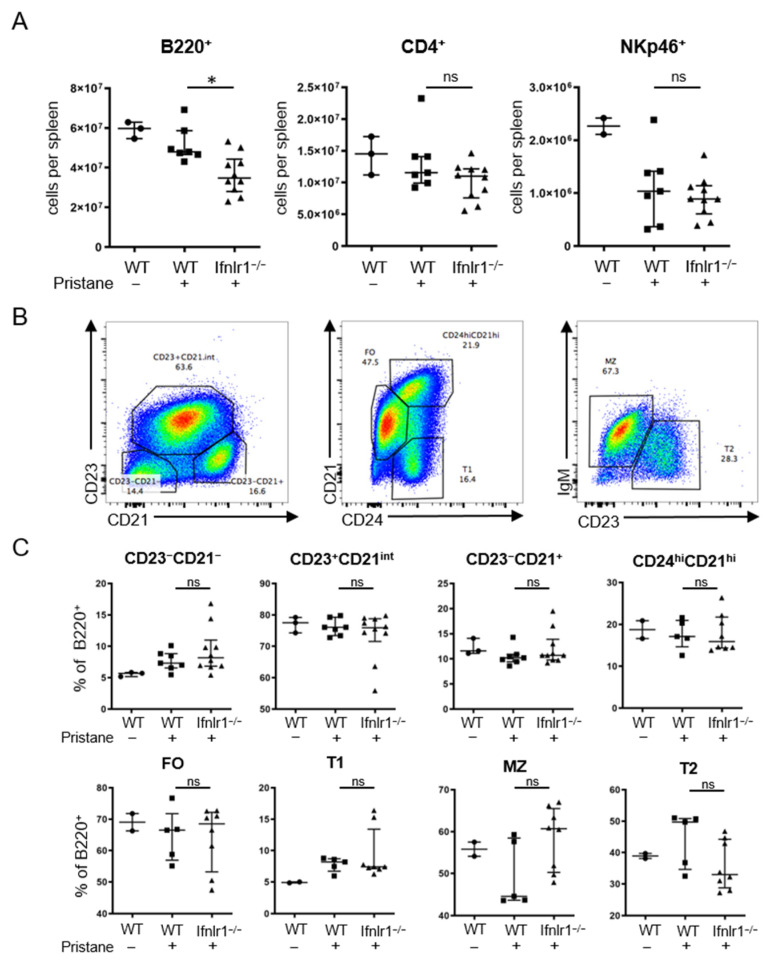
*Ifnlr1*^−/−^ mice show reduced numbers of B220^+^ cells in the spleen after pristane treatment. (**A**) Total cell numbers per spleen of B220^+^ B cells, CD4^+^ T cells and NKp46^+^ cells were determined by flow cytometry at 6–7 months after pristane treatment. (**B**) Gating strategy for the flow cytometric analysis of splenic B220^+^ B cell subsets. (**C**) Proportion of indicated populations among total splenic B220^+^ B cells. Each symbol represents an individual sample; bars represent median with interquartile range. For some mice, not enough tissue was available for flow cytometry. Statistical analyses to compare WT + pristane vs. *Ifnlr1*^−/−^ + pristane were performed using Mann–Whitney U test. * = *p* < 0.05; ns = not significant. Values of WT without pristane are shown as reference. Findings were confirmed in at least one other independent experiment.

Total cell numbers per spleen of B220^+^ B cells, CD4^+^ T cells and NKp46^+^ cells were determined by flow cytometry at 6 months after pristane treatment. Numbers of B220^+^ B cells were significantly reduced in *Ifnlr1*^−/−^ mice treated with pristane. No difference was found in regard to absolute numbers of CD4^+^ T cells and NKp46^+^ cells (Figure 5A). When further analyzing subgroups of B220^+^ B cells based on their surface expression of CD21, CD23 and CD24, no difference in subset compositions could be observed (Figure 5B,C). These data suggest that IFNLR1 deficiency affects the frequency of peripheral B cell populations while maintaining their phenotype in the context of induced systemic autoimmunity and interferonopathy. 

### 2.6. Ifnlr1^−/−^ Mice Show an Increased Proportion of Peripheral CD115^+^Ly6C^+^ Monocytes at 2 Weeks after Pristane Treatment

**Figure 6:**
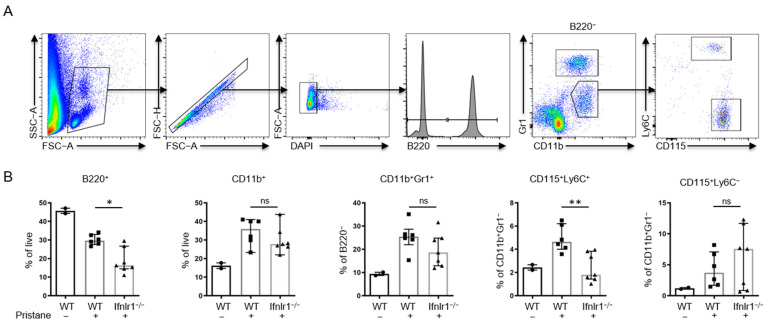
*Ifnlr1*^−/−^ mice show an increased proportion of peripheral CD115^+^Ly6C^+^ monocytes at 2 weeks after pristane treatment. (**A**) Gating strategy for the flow cytometric analysis of peripheral blood leukocytes. (**B**) Proportion of indicated populations among total living single cells or among indicated subpopulations. Each symbol represents an individual sample and all samples were included. Bars represent median with interquartile range. Bars represent group mean ± SD. Statistical analyses to compare WT + pristane vs. *Ifnlr1*^−/−^ + pristane were performed using Mann–Whitney U test. * *p* < 0.05, ** = *p* < 0.005, ns = not significant. Values of WT without pristane are shown as reference.

Mice were bled two weeks after pristane injections and peripheral blood mononuclear cells (PBMCs) were analyzed by flow cytometry. In both *Ifnlr1*^−/−^ and WT mice treated with pristane, a relative decrease in circulating B220^+^ B cells and a relative increase in CD11b^+^ cells and CD11b^+^Gr1^hi^ neutrophils were observed (Figure 6A,B). Interestingly, the proportion of CD115^+^Ly6C^+^ inflammatory monocytes among CD11b^+^Gr1^−^ cells was significantly reduced in pristane-treated *Ifnlr1*^−/−^ mice compared with pristane-treated WT controls (Figure 6A,B). This suggests that type III IFN contributes to the initiation of the inflammatory cascade at the very beginning of the development of pristane-induced systemic inflammation and autoimmunity in mice.

## 3. Discussion

It is widely accepted that type I IFNs play an important role in the pathogenesis of SLE: treatment of patients with IFN-α may cause drug-induced lupus, many patients with active SLE show an interferon signature of blood mononuclear cells, and lupus-prone mice treated with double-stranded RNA or an adenovirus encoding IFN-α develop a more severe disease [8,10,11,12,13,14,15,16,17]. The more recently discovered members of the interferon family, namely type III interferons (IFN-λ), were initially described to play a role in the defense against viral pathogens on barrier surfaces [24,46,47]. However, there is growing evidence that IFN-λ also contributes to inflammation in autoimmune disorders [48]. This includes findings of increased concentrations of type III IFN in the sera of patients with SLE, which correlated with disease activity [25,26,27,28,29]. In a TLR7-induced lupus mouse model, *Ifnlr1*-deficient mice displayed reduced skin and kidney inflammation as well as decreased immune complex deposition [30]. 

Here, using a murine model of SLE that is not dependent on skin inflammation, we demonstrate that type III IFN plays an important role in controlling autoimmune and inflammatory responses. We used the pristane-induced lupus model in C57BL/6 mice carrying an intact *Mx1* allele (B6.A2G-*Mx1*) [49,50,51,52] and B6.A2G-*Mx1*-*Ifnlr1*^−/−^ mice deficient in functional IFN-λ receptors [53]. Survival rates after pristane injection were likely reduced due to the wild type Mx1 allele and were improved by *Ifnlr1* deficiency. Lipogranuloma formation was reduced by *Ifnlr1* deficiency while not being completely suppressed. Having defective IFN-λ receptors resulted in significantly reduced titers of anti-dsDNA antibodies in the early phase of the disease, but not at a later stage. This could indicate that type III IFN pathways play an indirect role in autoantibody generation, and that their absence delays the development of autoantibodies without completely preventing it.

To further corroborate these findings, we quantified the numbers of dsDNA-specific antibody-secreting cells in the kidneys, spleens and bone marrow, which are known to be the primary sites of autoantibody production in NZB/W F1 mice. While our findings did not reach statistical significance, presumably because of insufficient numbers of pristane-treated mice, there was a clear trend towards reduced numbers of IgG-secreting cells in the kidneys and of dsDNA-specific antibody-secreting cells in the bone marrow of *Ifnlr1*^−/−^ mice. 

The different types of lupus nephritis with subsequent injury of the kidney have in common that they are associated with the deposition of autoantibodies within the glomeruli. These depositions of immune complexes are found in most patients with lupus nephritis; however, renal immune complex formation is not sufficient to cause renal inflammation and glomerular damage [54]. Whereas it has been traditionally assumed that circulating immune complexes deposit within the glomeruli during the filtration process (circulating immune complex hypothesis), there is accumulating evidence that circulating autoantibodies bind in situ to local nuclear antigens (planted antigen hypothesis) [55,56,57,58,59,60]. An emerging concept is also the local production of autoantibodies by renal plasma cells [61]. The origin of renal plasma cells remains to be elucidated. Presumably, migratory plasma blasts are attracted by chemokines such as CXCL-12 and migrate from the blood into the inflamed kidneys. In addition, plasma cells could differentiate within inflammation-induced tertiary lymphoid organs, which are detectable within inflamed kidneys, both in lupus patients as well as mouse models [62,63]. Previously, we described that the majority of renal plasma cells produce autoantibodies against dsDNA or nucleolin, at least in female NZB/W F1 mice [61].

We found the deposition of IgG immune complexes in most of the kidneys of mice treated with pristane, independently of IFN-λ receptor deficiency (data not shown). The presence of immune complexes in the glomeruli induces a cascade of inflammatory reactions, with the activation of the complement system, the activation of both immune and renal epithelial cells, the release of pro-inflammatory cytokines, the expression of adhesion molecules by endothelial cells and the recruitment of immune cells (B and T cells, macrophages, DCs) from the periphery [64].

Previous work has shown that Lineage^−^CD11b^hi^CD11c^low^F4/80^hi^ macrophages play a key role in poly(I:C)-mediated aggressive lupus glomerulonephritis [17]. In contrast, homeostatic kidneys harbor an F4/80^hi^CD11b^low^ tissue-resident macrophage population that functions to scavenge circulating immune complexes [65]. Importantly, we found that *Ifnlr1* deficiency promoted the numbers of CD11b^low^F480^hi^ tissue-resident macrophages, reproducing the state that we observed in untreated control mice. Absolute numbers of kidney leukocytes, including neutrophils, Lineage^−^CD11b^hi^CD11c^lo^ mononuclear phagocytes and CD4^+^ T cells, were comparable between WT and *Ifnlr1*-deficient mice. These data suggest that a lack of type III IFNs promotes immune homeostasis in autoimmune-prone hosts.

Until now, the role of natural killer cells and other innate lymphoid cells in SLE or, more precisely, in lupus nephritis was not clear. There is, however, indirect evidence for their involvement in the pathological chain of events [66,67,68,69,70]. We found that NKp46^+^Eomes^+^Lineage^−^ conventional natural killer cells (cNK) were significantly increased in the kidneys of pristane-treated mice, a finding that was reversed in *Ifnlr1*-deficient mice. No difference in the numbers of NKp46^+^Eomes^−^ Lineage^−^ ILC1s was observed. Moreover, the proportion of ILC1s and cNK within the population of Lineage^−^NKp46^+^ shifted to higher relative numbers of cNK cells in the kidneys of pristane-treated mice, while in *Ifnlr1*-deficient mice treated with pristane, the abundance of these cell populations was similar to levels observed in untreated control mice. ILC1s are thought to be tissue-resident, whereas both circulating and tissue-resident populations of cNK cells have been described in lymphoid organs, in the salivary glands and in the liver [71,72,73,74]. This suggests that recruiting cNK cells from the periphery to the kidney in the context of an immune-complex-mediated inflammatory response is at least partially regulated by type III IFN signaling. 

In our study, none of the pristane-treated mice developed proteinuria within 6 months after pristane injection (data not shown). This is in line with studies showing that renal affection is rather mild in pristane-induced lupus on a C57Bl/6 background [36,37,75]. In line with the absence of proteinuria, we could not detect structural damage in PAS-stained kidney sections (data not shown). 

Although renal inflammation was not as severe as in the NZB/W F1 or Mrl/lpr lupus mouse models, our data clearly demonstrate that the numbers of inflammatory monocytes and cNK cells in response to immune complexes are controlled by type III IFN. 

These findings strengthen the notion that IFN-λ promotes the loss of homeostasis and innate inflammatory responses that, in turn, results in a pro-inflammatory environment, potentially promoting increased plasma cell homing in the kidneys of autoimmune hosts. 

Finally, we found that, two weeks after pristane injection, the numbers of circulating CD115^+^Ly6C^+^ monocytes were reduced in *Ifnlr1*-deficient mice. This indicates that, in the early phase of disease induction, type III IFNs play an additive role to type I IFNs, which have been described as the main drivers of pathogenicity in human SLE in general, as well as specifically in pristane-induced murine lupus. 

To conclude, the present study supports a regulatory role for type III IFN in pristane-induced murine autoimmunity, and in systemic and renal inflammation. Type III IFN signaling is not necessary for the development of autoantibodies, but it seemingly contributes to and accelerates their development. Type III IFN signaling appears to affect the renal microenvironment in such a way that conventional NK cells are recruited from the periphery and monocytes/macrophages show distinct phenotypes. Further studies are needed to mechanistically tie these findings together and extrapolate them to human SLE, potentially paving the way for new therapeutic targets. 

## 4. Materials and Methods

### 4.1. Mice and Animal Housing

Mice were housed under standard pathogen-free conditions (12 h light–dark cycle, temperature 20 °C–20.5 °C, rel. humidity 49–52%) in a facility of the University of Freiburg. Animal experiments were approved by the local authorities (*Regierungspräsdium Freiburg*, G-15/164, G-17/122). We used C57BL/6 mice carrying an intact *Mx1* allele that attributes resistance to certain myxoviruses including influenza A and for which most inbred strains of laboratory mice show large deletions or nonsense mutations [49,50,51,52,76]. We used B6.A2G-*Mx1*-*Ifnlr1*^−/−^ (*Ifnlr1*^−/−^ mice) and B6.A2G-*Mx1-Ifnlr1*^+/+^ (WT mice). Breeding and genotyping was performed as previously described [53,77]. No difference in the parameters described above could be found in *Ifnlr1*^−/−^ mice and WT mice in the absence of pristane-induced autoimmunity. 

### 4.2. In Vivo Intraperitoneal Injections

Mice were manually fixed, inclined to 45° with head down and 500 µL pristane (2,6,10,14-tetramethylpentadecane, TMPD, Sigma-Aldrich, P2870) was slowly injected into the upper abdomen. Control mice were injected with the equivalent volume of phosphate-buffered saline (PBS).

### 4.3. Collection Peripheral Blood and Serum

To obtain peripheral blood, the vena facialis was punctured using a 20 or 22 G needle and a few drops of blood were either collected in heparin-containing tubes for FACS analysis or into a Microtainer^®^ for serum preparation. The latter was left at room temperature (RT) for 30–60 min and then spun down at 104 g. Serum was transferred to a fresh microtube (Eppendorf^®^) and stored at −80 °C until use.

### 4.4. Euthanasia of Mice and Harvesting of Organs

Mice were euthanized either by cervical dislocation after inhalative anesthesia with Isoflurane^®^ or by CO_2_. After testing for negative pain reaction and reflexes, the fur was disinfected with 70% alcohol before abdominal and thoracic cavities were opened. For intracardial perfusion, the inferior vena cava was sectioned and 10–20 mL of ice-cold PBS was injected into the left ventricle until the kidneys turned pale. Kidneys and spleen were harvested, weighed and stored on ice swimming in PBS until further processing. Tibia and femur bones were harvested and placed in PBS on ice for isolation of bone marrow cells. 

### 4.5. Semi-Quantitative Scoring of Peritoneal Lipogranulomas

After opening the abdominal cavity, peritoneal lipogranuloma formation was assessed using a semi-quantitative score: 0 = none; 0.5 = very low; 1 = low; 1.5 = moderate and 2 = high. 

### 4.6. Quantification of Total Serum IgG Levels

First, 96-well MaxiSorp™ plates were coated with anti-mouse IgG (2 µg/mL in 50 µL PBS per well) overnight at 4 °C, washed three times with PBST (0.05% Tween 20 (Sigma-Aldrich, St. Louis, MO, USA; P7949-500) in PBS), blocked with 150 µL blocking solution per well for 30 min at room temperature and washed with PBST. Serum was diluted as indicated in PBS with 2% fetal calf serum (FCS) and 50 µL of the diluted serum was added per well and incubated at RT for 2 h. After washing with PBST, secondary HRP goat anti-mouse IgG Fc-γ (Jackson, Bar Harbor, ME, USA; 115-006-008) was added at a dilution of 1:5000 (50 µL per well) and incubated for 1 h at RT. After washing with PBST, 100 µL of ABTS solution (Roche, Basel, Switzerland; 11-684-302-001) was added per well and OD was measured using an ELISA reader. 

### 4.7. Quantification of Serum Anti-dsDNA Levels

First, 96-well MaxiSorp™ plates were pre-coated with 20 µg/mL poly-L-lysine (Sigma-Aldrich P4707) in 50 µL TE-buffer (10 mM Tris/HCl; 1 mM EDTA; pH 7.4) per well and incubated overnight at 4 °C. After washing with TE-buffer, 50 µL of TE-buffer containing 20 µg/mL of deoxyribonucleic acid sodium salt from calf thymus (Sigma-Aldrich, D4522-5 mg) was added per well for coating overnight at 4 °C. Further steps were carried out as described above for total IgG-ELISA. 

### 4.8. Quantification of Antibody Secreting Cells

For detection of IgG-secreting cells, MultiScreenHTS-IP ELISpot plates (Merck, Darmstadt, Germany) were pre-wetted with 30 µL/well of 70% ethanol for 1 min followed by three washing steps. Next, the plates were coated with 100 µL of a 2 µg/mL goat anti-mouse IgG antibody (H+L) solution diluted in PBS overnight at 4 °C. The next day, plates were washed four times and unspecific binding sites were blocked with 150 µL/well PBS/2% FCS for at least 1 h at room temperature or overnight at 4 °C. Cells were then incubated at appropriate cell numbers for 2 h at 37 °C with 5% CO_2_ followed by washing five times. Next, secreted IgG molecules were detected with 100 µL/well Fc γ-chain-specific anti-IgG antibody coupled to horseradish peroxidase molecules diluted in PBS/2% FCS for 1 h at RT. After four washing steps, 100 µL/well of the substrate TMB (KPL, 50-77-03) was added and the enzyme substrate reaction was stopped by multiple washing steps with H2O from both sides of the plate after first spots were visible. Plates were dried overnight, protected from light, and were scanned the next day. All washing steps were performed with 150 µL/well of PBS. 

### 4.9. Preparation of Kidney Tissue for Flow Cytometry

After removal of the capsule, kidneys were cut into small pieces using scissors and a scalpel and placed into a C-Tube™ (Miltenyi Biotech, Bergisch Gladbach, Germany; 130-096-334) containing 10 mL of a digestion mix (1.5 mg/mL collagenase D, 200 IE/mL DNase I, 2% FCS in DMEM or HBSS). After homogenization by using the GentleMacs™ (Miltenyi Biotech, (program: *m_lung_01*), incubation in the digestion mix at 37 °C for 30 min and further homogenization, (GentleMacs™, program *spleen_04*), the digested tissue was filtered through a 70 µm cell strainer into a 50 mL tube. The single cell suspension was centrifuged at 800× *g* for 10 min at 4 °C, and the pellet was either resuspended in red cell lysis buffer (RCLB: 500 mL ddH2O, 150 mM NH4CL, 29 mM HEPES, 0.1 mM EDTA pH 8.0) or in 8 mL of 45% Percoll (Sigma-Aldrich, St. Louis, MO, USA; P1644-1L) whenever a Percoll gradient was performed. In this case, the kidney tissue was gently layered onto 3 mL of 80% Percoll, which had been added to a 15 mL tube. The Percoll gradient was centrifuged at 1350× *g* for 20 min at room temperature, the brake of the centrifuge being programmed to its lowest level. Using a 3 mL Pasteur pipette, the dead cells and debris at the top were aspirated and the leukocytes at the interface of the two Percoll concentrations were carefully collected with a clean Pasteur pipette and transferred to a new 15 mL tube. This tube was filled with PBS or FACS buffer (2% FCS and 1% EDTA 0.5 mM in PBS), inverted several times to mix the remaining Percoll and finally centrifuged at 2000 rpm (800× *g*) for 10 min at 4 °C. Whenever a Percoll gradient was not performed, the cell pellets were re-suspended in 1 mL RCLB and incubated at room temperature for 4–5 min. At least 9 mL of ice-cold PBS or FACS buffer was added to sufficiently dilute the RCLB. After centrifugation, cells were re-suspended in FACS buffer and transferred to a 96-well plate.

### 4.10. Preparation of Spleen Tissue for Flow Cytometry

Spleens were minced using scissors and a scalpel on a petri dish and transferred to a C-tube™ containing 10 mL of ice-cold PBS and homogenized using the GentleMacs™ (program *spleen_04*) and finally transferred to a 50 mL tube passing through a 70 µm cell strainer. Alternatively, whole spleen was pushed through a 70 µm cell strainer and flushed into a 50 mL tube with ice-cold PBS. Single cell suspensions were centrifuged at 500× *g*, supernatant was removed and pellet was re-suspended in 1 mL RCLB. After 5 min at room temperature, at least 9 mL of PBS was added to dilute RCLB and the cell suspension was centrifuged again. This step was repeated when necessary. The cells were re-suspended in the remaining 100–200 µL and transferred to a 96-well plate or a FACS tube. 

### 4.11. Preparation of Bone Marrow Single Cell Suspensions

Femur and tibia were separated from muscle and other tissues before bones were sectioned at distal and proximal ends. Using a 24 G needle and 5 or 10 mL syringes, bones were flushed with 5 mL of ice-cold PBS and bone marrow was collected into a 50 mL tube. Cell suspensions were aspirated up and down through a 20G needle in order to break apart clumps and finally passed through a 70 µm cell strainer into a fresh 50 mL tube for further processing. 

### 4.12. Preparation of Peripheral Blood Cells

Heparinized blood was centrifuged at 500× *g* for 5 min, supernatant was removed and red cell lysis was performed. At least 500 µL of PBS or FACS buffer was added after 5 min to dilute RCLB. The cell suspension was centrifuged again at 500× *g* for 5 min before the cell pellet was re-suspended with the desired volume of FACS buffer and transferred to a 96-well plate or a FACS tube.

### 4.13. Flow Cytometry of Single Cell Suspensions

Single cell suspensions of kidneys, spleen or peripheral blood were prepared as described above, centrifuged at 500–650× *g* for 5 min before re-suspending in 50–100 µL blocking buffer and incubating on ice for 30 min. After centrifugation, the cell pellet was re-suspended in 50–100 µL antibody mix and incubated on ice in the dark for at least 30 min. After incubation, two washing steps with 100 µL FACS buffer were performed. Whenever the antibody mix contained biotinylated antibodies, Streptavidin labeled with V500™ (BD Bioscience, Franklin Lakes, NJ, USA; 561419) was added in a 1:800 dilution and incubated on ice, in the dark, for 30 min. After two washing steps, the cell suspension was centrifuged one last time and the pellet re-suspended in 200–300 µL FACS buffer containing DAPI (Life Technologies, Carlsbad, CA, USA; D3571) in a dilution of 1:2000. After transfer to FACS tubes, the live single cell suspensions were analyzed by flow cytometry. For intracellular staining, PBS was used for two washing steps instead of FACS buffer and the pellet was re-suspended in 100 µL PBS containing Fixable Viability Dye™ (eBioscience, Frankfurt am Main, Germany; 65-0863-14) in a dilution of 1:1000 and incubated on ice, in the dark, for another 30 min. After washing twice with PBS, the pellet was re-suspended in 100 µL fixation buffer and kept at 4 °C overnight for a maximum of 18 h. Fixed cells were centrifuged the next day at 570× *g* for 7 min and washed twice with permeabilization buffer (Permeabilization Buffer 10×, eBioscience, diluted 1:9 in purified water). Intracellular antibodies in 50–100 µL FACS buffer were added and incubated at 4 °C in the dark for 90 min. The following antibodies were used: B220 (CD45R) (clone: RA3-6B2, dilution 1:400, eBioscience, 47-0452-82); CD115 (clone: c-fms, dilution 1:100, eBioscience, 17-1152-82); CD11b (clone: M1/70, dilution 1:800, eBioscience, 25-0112-82); CD11c (clone: N418, dilution 1:400, eBioscience, 45-0114-80); CD19 (clone: MB19-1, dilution 1:200, eBioscience, 13-0191-81); CD3 (clone: 145-2C11, dilution 1:400, eBioscience, 13-0031-86); CD45 (clone: 30-F11, dilution 1:200, eBioscience, 47-0451-82); CD45.2 (clone: 104, dilution: 1:200, eBioscience, 47-0454-82); CD21/CD35 (clone: 7E9, dilution 1:200, Biolegend, 123409); CD23 (clone: B3B4, dilution 1:200, BioLegend, 101607); CD24 (clone: M1/69, dilution 1:200, BioLegend, 101807); IgM (clone: RMM-1, dilution 1:300, BioLegend, 406507); CD5 (clone: 53-7.3, dilution 1:200, eBioscience, 13-0051-82); Eomes (clone: Dan11mag, dilution 1:100, eBioscience, 50-4875-82); F4/80 (clone: Cl:A3-1, dilution 1:100, AbD Serotec, MCA497APCT); Gr1 (clone: RB6-8CJ, dilution 1:100), eBioscience, 11-5931-85); Isotype control (clone: eBR2a, 1:100, eBioscience, 50-4321-80); Ly6C (clone: HK1.4, dilution 1:100, eBioscience, 45-5932-82); NK1.1. (clone: PK136, dilution 1:200, eBioscience, 25-5941-81); NK1.1 (clone: PK136, dilution 1:200, BioLegend, 108703); Sca1 (clone: D7, dilution 1:400, eBioscience, 25-5981-81); NKp46 (clone: 29A1.4, dilution 1:100, eBioscience, 13-3351-82); NKp46 (clone: 29A1.4, dilution 1:100, eBioscience, 46-3351-82). After washing twice with FACS buffer, cells were finally transferred from the 96-well plates to FACS tubes and analyzed by flow cytometry on a FACS BD CantoTM II or BD LSRFortessaTM (BD Bioscience, Franklin Lakes, NJ, USA). For compensation with single stained controls, either a single cell suspension of spleen cells or 50 µL of UltraComp beads™ was incubated with the respective antibodies. Whenever possible, the same antibodies as in the master stain were used; otherwise, anti-CD4 or anti-CD8 antibodies with same fluorochromes were used. Compensation was calculated using FACS DivaTM or FlowJo SoftwareTM (BD Bioscience, Franklin Lakes, NJ, USA). Data were analyzed with FlowJoTM v9 and v10. 

### 4.14. Statistical Analysis

Means, medians and interquartile ranges were calculated using Excel^®^ or Graphpad Prism^®^. As a Gaussian distribution was not evident, non-parametric Mann–Whitney U test was applied in GraphPad Prism 9 to test for the null hypothesis between pristane-treated *Ifnlr1*^−/−^ mice and pristane-treated WT mice. Untreated control mice were included merely as a reference. The effects of pristane on WT mice has been well described before [35,37].
